# Environmental and Biological Impact of Fly Ash and Metakaolin-Based Alkali-Activated Foams Obtained at 70°C and Fired at 1,000°C

**DOI:** 10.3389/fchem.2022.845452

**Published:** 2022-03-09

**Authors:** Cristina Leonelli, Janez Turk, Giovanni Dal Poggetto, Michelina Catauro, Katja Traven, Alenka Mauko Pranjić, Vilma Ducman

**Affiliations:** ^1^ Department of Engineering “Enzo Ferrari”, University of Modena and Reggio Emilia, Modena, Italy; ^2^ Slovenian National Building and Civil Engineering Institute (ZAG), Ljubljana, Slovenia; ^3^ Department of Engineering, University of Campania “Luigi Vanvitelli”, Aversa, Italy

**Keywords:** alkali-activated foams, fly ash, metakaolin, Na-based activator, K-based activators, LCA, leaching, ecotoxicity

## Abstract

Alkali-activated foams (AAFs) are inorganic porous materials that can be obtained at temperatures well below 100°C with the use of inorganic wastes as aluminosilicate precursors. In this case, fly ash derived from a Slovenian power plant has been investigated. Despite the environmental benefits *per se*, due to saving of energy and virgin materials, when using waste materials, it is of extreme importance to also evaluate the potential leaching of heavy metal cations from the alkali-activated foams. This article presents an environmental study of a porous geopolymer derived from this particular fly ash, with respect to the leachability of potentially hazardous elements, its environmental toxicity as determined by biological testing, and the environmental impact of its production. In particular, attention was focused to investigate whether or not 1,000°C-fired alkali-activated fly ash and metakaolin-based foams, cured at 70°C, are environmentally friendlier options compared to unfired ones, and attempts to explain the rationale of the results were done. Eventually, the firing process at 1,000°C, apart from improving technical performance, could reinforce heavy metal cation entrapment within the aluminosilicate matrix. Since technical performance was also modified by addition of different types of activators (K-based or Na-based), as well as by partial replacement of fly ash with metakaolin, a life cycle assessment (LCA) analysis was performed to quantify the effect of these additions and processes (curing at 70°C and firing at 1,000°C) in terms of global warming potential. Selected samples were also evaluated in terms of leaching of potentially deleterious elements as well as for the immobilization effect of firing. The leaching test indicated that none of the alkali-activated material is classified as hazardous, not even the as-received fly ash as component of new AAF. All of the alkali-activated foams do meet the requirements for an inertness. The highest impact on bacterial colonies was found in samples that did not undergo firing procedures, i.e., those that were cured at 70°C, which induced the reduction of bacterial *Enterococcus faecalis* viability. The second family of bacteria tested, *Escherichia coli*, appeared more resistant to the alkaline environment (pH = 10–12) generated by the unfired AAMs. Cell viability recorded the lowest value for unfired alkali-activated materials produced from fly ash and K-based activators. Its reticulation is only partial, with the leachate solution appearing to be characterized with the most alkaline pH and with the highest ionic conductivity, i.e., highest number of soluble ions. By LCA, it has been shown that 1) changing K-based activators to Na-based activators increases environmental impact of the alkali-activated foams by 1%–4% in terms of most of the impact categories (taking into account the production stage). However, in terms of impact on abiotic depletion of elements and impact on ozone layer depletion, the increase is relatively more significant (11% and 18%, respectively); 2) replacing some parts of fly ash with metakaolin also results in relatively higher environmental footprint (increase of around 1%–4%, while the impact on abiotic depletion of elements increases by 14%); and finally, 3) firing at 1,000°C contributes significantly to the environmental footprint of alkali-activated foams. In such a case, the footprint increases by around one third, compared to the footprint of alkali-activated foams produced at 70°C. A combination of LCA and leaching/toxicity behavior analysis presents relevant combinations, which can provide information about long-term environmental impact of newly developed waste-based materials.

## Introduction

Alkali-activated materials (AAMs) are aluminosilicate-based binders that, after activation, harden at room or slightly elevated temperatures, i.e., below 80°C (Palomo et al., 2021; Provis et al., 2014). In recent years, AAMs have gained a lot of attraction due to the fact that waste materials can be utilized for their production and that they could compete with concrete or ceramic in certain applications (Provis, 2018a). In the most general description, AAMs are inorganic systems consisting of two main components: a reactive solid component mainly composed of amorphous aluminosilicate, also called precursor, and an alkaline activator solution, the activator, where different alkali hydroxides and silicates, as well as carbonates and sulfates, come into consideration (Provis and van Deventer, 2013). There are many kinds of aluminosilicate source suitable to be used such as metakaolin (also the most studied one) (Clausi et al., 2016), kaolin (Balczar et al., 2016; Esaifan et al., 2015; Sgarlata et al., 2021), calcinated clays (Provis, 2018b), fly ashes (Rakngan et al., 2018), various slags (Adesanya et al., 2017; Češnovar et al., 2019), different types of mineral wool (Pavlin et al., 2021), volcanic ash (Djobo et al., 2016), and red muds (Badanoiu et al., 2015). The microstructure and therefore the overall performance (mechanical properties, the durability of the material) of the alkali-activated binder is strongly dependent on various factors such as type and amount of precursor, particle size distribution, presence of amorphous phase, Na(K)/Si/Al ratio, type and concentration of the activator, the curing regime, etc. (Duxson et al., 2005; Williams and van Riessen, 2010; Traven et al., 2019; Češnovar et al., 2019).

Concerning all the facts regarding AAM production, they could be designed to have superior properties compared to conventional cementitious as well as ceramic materials, and thus, the range of final products/applications that can be obtained by the alkali activation process is also very broad (e.g., paving stones, blocks, curbs, foams, slabs, partitions, refractory materials, and materials for specific industrial applications). Especially, alkali-activated foams (AAFs) seem to be promising because they can be obtained at temperatures well below 100°C but offering performance similar to foamed glass or aerated concrete (Ducman and Korat, 2016). Porosity is introduced by different foaming agents, like hydrogen peroxide (Masi et al., 2014; Abdollahnejad et al., 2015), perborate, (Korat and Ducman, 2020), and metals (Masi et al., 2014; Hlavacek et al., 2015; Kamseu et al., 2015), which decompose or react in alkaline mixture, forming gasses at the proper stage of fresh paste induration to create a porous structure. Density as low as 0.3 g/cm^3^ could be achieved by this method, whereas porosity significantly influences mechanical performance. A thermal post-treatment of the AAFs by exposing such foams to high temperature between 800°C and 1,000°C can be implemented without affecting density too much (Monich et al., 2018; Bajare et al., 2019; Traven et al., 2021).

When it comes to the potential application of AAM, foams in the present case, it is of extreme importance to make an assessment of the environmental influence of newly developed products. Environmental assessment is a broad concept consisting of different biological, chemical, and physical parameters, such as ecotoxicity, leaching behavior, and radioactivity. Environmental assessment can be conducted also by means of life cycle assessment (LCA), which is a standardized method to quantify and, thus, compare the sustainability of different materials (ISO 14040:2006; ISO 14044:2006).

Concerning the ecotoxicity aspects of this study, we need to consider the potential environmental impact caused by the leaching of excessive alkali that may increase the solubility of some amphoteric oxides. A careful evaluation of the effect on pH, ionic conductivity, and metals characterizing the eluates from each formulation is the first step to determine the structural stability of the AAMs (Lancellotti et al., 2021). During the service life of the AAMs, the contact with live organism is possible; thus, the definition of possible damages on that front should also be estimated. Only few studies have already been performed regarding antibacterial and/or cytotoxicity aspects of AAM (Catauro et al., 2017, 2021; Rubio-Avalos, 2018). In one of the latest (Sgarlata et al., 2021) investigated bioenvironmental impacts of metallurgical slags and stone wool-based AAMs, the authors evidenced the absence of ecotoxicity in terms of heavy metal cation release as well as in terms of bio-impact. Nevertheless, the eventual presence of an antimicrobial functionality in these materials is not necessarily a negative issue, since some metakaolin-based geopolymers with antimicrobial properties have been specifically developed for water purification applications (O’Connor et al., 2010).

Additionally, LCA provides information on how certain materials or processes contribute to the environmental impacts, one of the most important being the global warming potential (GWP). Calculation ideally takes into account different stages of a product’s life cycle, such as raw material extraction, material processing and manufacturing of the product, its installation, use, maintenance, and, finally, product end-of-life treatment (re-use, recycling, and/or final disposal) (European Commission - Joint Research Centre. Institute for Environment and Sustainability, 2010; Curran, 2013). For alkali-activated products, on general, it is known that in comparison to concrete or ceramics products, significant environmental benefits are to be expected (Provis et al., 2010; Gervasio and Dimova, 2018; Kvočka et al., 2020). For example, Jiang et al. (2014) compared the cradle-to-gate greenhouse gas emissions (GHG), energy use, water use, and potential environmental toxicity of conventional, glass powder, and alkali-activated slag (AAS) concrete and mortar. The authors concluded that using alternative cementitious materials as cement replacements could significantly reduce the environmental impacts of cement-based products (e.g., AAS concrete had 73% lower GHGs, 43% less energy, and 25% less water). In another study, Bianco et al. (2021) compared the AAM produced with a silicate activator derived from waste glass (AABR) or with commercially available chemicals (AABC) to ordinary portland cement (OPC). The authors showed that the adoption of alkali-activated concretes instead of OPC concrete allows a significant reduction in environmental categories of global warming potential (64% and 70% reduction for AABC and AABR, respectively), acidification potential (23% and 35% reduction for AABC and AABR, respectively), and terrestrial eutrophication (53% and 60% reduction for AABC and AABR, respectively).

The aim of the present study was to assess environmental performance of AAF whose technical properties are described in paper by Traven et al. (2021). The high porosity of such foams could be critical in terms of cation exchange with natural environment; thus, an accurate bio-impact is beneficial to ascertain their safe use and durability. The assessment consisted of an evaluation of environmental impacts arising from manufacturing of the AAF and an evaluation of leaching parameters affecting ecotoxicity. The former evaluation was conducted by means of LCA of different environmental indicators. Additionally, the second novelty of this study was the experimentation on the toxicity of fly ash (FA)-based geopolymers by studying the response of bacterial and cell growth.

## Experimental

### Materials

For the alkali activation, two types of powder precursors were used, FA obtained from a Slovenian thermal plant and metakaolin (MK) from company Argeco, Fumel, France. The main constituents of the fly ash are as follows: 44.8 wt% SiO_2_, 22.9 wt% Al_2_O_3_, 12.4 wt% CaO, and 10.7 wt% Fe_2_O_3_. The main constituents of the metakaolin are as follows: 68.2 wt% SiO_2_, 25.3 wt% Al_2_O_3_, and 2.2 wt% Fe_2_O_3_. Activators were chosen from sodium silicate (chemical formula: Na_2_SiO_3_, SiO_2_:Na_2_O = 1.97 mass%; 54.2 mass% aqueous solution) Crystal 0112 produced by Tennants Distribution, Ltd., Manchester, United Kingdom; potassium silicate (chemical formula: K_2_SiO_3_, SiO_2_:K_2_O = 1.63 mass%; 51.4 mass% aqueous solution) Betol K 5020 T produced by Woellner Austria GmbH, Judendorf-Straßengel, Austria; and NaOH and KOH 41.7 mass% water solutions (both produced by Donau Chemie, Wien, Austria). A solution of hydrogen peroxide, H_2_O_2_, with a concentration of 30 vol% was used as the foaming agent (produced by Carlo Erba Reagents, Cornaredo, Italy) and sodium dodecyl sulphate (SDS; produced by Acros organics) as a surfactant. Mixtures were developed and presented in detail in a previous paper by Traven et al. (2021), and compositions of samples selected for this study are given in Table 1. All lightweight alkali-activation foams were foamed with 1 mass% of H_2_O_2_, and pores were stabilized with the addition of 1 mass% of sodium dodecyl sulfate as a surfactant. The fresh foamed pastes were poured into 20 × 20 × 80 mm^3^ molds and cured at 70°C for 3 days. The hardened AAFs were then exposed to elevated temperatures, and the samples exposed to 1,000°C were selected for this study. The first part of sample designations is listed in Table 1, whereas the designations in the continuation of this paper are composed of the first part and the exposure temperature (for example: FA73Na S.T. is a sample of composition FA73Na and exposed to room temperature, whereas FA73Na 1,000 is a sample of composition FA73Na and exposed to 1,000°C). Therefore, in general, S.T. in the designation stands for room temperature, 1,000 for 1,000°C, and Na or K refers to the activator type used.

**TABLE 1:** List of compositions of the different alkali-activation foams prepared for the study (all in mass%).

Sample designation	FA	MK	Na_2_SiO_3_	K_2_SiO_3_	NaOH	KOH	H_2_O_2_	SDS
FA73Na	69.0	–	23.5	–	5.5	–	1.0	1.0
FA36Na	34.5	34.5	23.5	–	5.5	–	1.0	1.0
F67K	66.9	–	–	28.9	–	2.1	1.0	1.0
FA33K	33.5	33.5	–	28.9	–	2.1	1.0	1.0

### Test Methods

#### Integrity Test

One of the first test adopted to demonstrate the quality of the chemical resistance of the AAM formulation is a rapid, yet substantial, qualitative test often adopted in literature (Ruiz-Santaquiteria et al., 2011). MilliQ water in the proportion of 1:100 solid/water ratio was used to bulk sample amounts (*w*_0_), ranging from 1.51 to 2.34 g. After 24 h, the water was removed, and the integrity was evaluated by estimating the following: 1) final pH solution, 2) smoothness, 3) hardness and finger pressure, and 4) water transparency.

#### pH and Ionic Conductivity Measurements

A more quantitative test to assess the chemical stability of the AAM after consolidation is proposed here. MilliQ water now in the proportion of 1:10 solid/water ratio was added to the ground and sieved (*d *< 125 µm) samples to test their chemical stability. After shaking the solution, a time was waited in order to sediment the solids prior to analyses. Ionic conductivity and pH measurements were performed with (respectively, Crison GLP31 and Crison GLP21, Hach Lange S.L.U., Barcelona, Spain) at different times: *t*_1_ = 0 h, *t*_2_ = 5 min, *t*_3_ = 10 min, *t*_4_ = 20 min, *t*_5_ = 2 h, *t*_6_ = 4 h, *t*_7_ = 6 h, *t*_8_ = 24 h, and *t*_9_ = 48 h, respectively.

#### Leaching Test

The ability of leaching heavy metals of all hardened samples was carried out according to the EN 12457 European standard “Characterization of waste–Leaching–Compliance test for leaching of granular waste materials and sludge.” The samples, crushed and sieved to particle sizes less than 4 mm, were placed in bi-distilled water with 10 l/kg liquid/solid weight ratio and maintained for 24 h. After the extraction and filtration of the leachates, the heavy metal ion’s concentrations were determined by ICP (ICPE-9000 Shimadzu, Tokyo, Japan). Before ICP analysis, the samples were acidified with HNO_3_ solution to pH = 2.

#### Antibacterial Test

In order to estimate the antibacterial properties of the AAF, gram negative/*Escherichia coli* (ATCC 25922) and gram positive/*Enterococcus faecalis* (ATCC 19433) were grown in the absence and presence of 50, 100, and 150 mg of the samples. Samples used for this analysis were ground to coarse powders that were radiated by UV light for 1 h in order to reach complete sterilization. The bacterial suspension of 10^5^ CFU/ml was obtained by diluting the strains in distilled saline water (4.5 mg/ml). After plating *E. coli* in TBX Agar medium (TBX Agar is a chromogenic selective medium used for the isolation and identification of *E. coli* according to ISO 16649-1, -2, and -3. Composed of enzymatic digest of casein, bile salt no. 3, and x-glucuronide mixed in agar, it has a final pH of 7.2 ± 0.2 at 25°C (produced by Liofilchem, Italy). After plating *E. faecalis* on Slanetz Bartley agar base (it is a selective medium for detection and enumeration of *enterococci* in water and other materials, according to ISO 7899-2). Composed of tryptose, yeast extract, glucose, dipotassium hydrogen phosphate, and sodium azide mixed in agar, it has a final pH of 7.2 ± 0.2 at 25°C (produced by Liofilchem, Italy). The samples were placed in the middle of Petri dishes. *E. coli* and *E. faecalis* dishes were incubated at 44°C for 24 h and at 36°C for 48 h, respectively. The diameter of inhibition halos (IDs) in relation to Petri dish diameter (DD = 6 cm) was calculated. Four measurements for each sample were carried out. Results are expressed as follows:

Bacterial Viability (in percentage) = [(DD − IDs) / (DD)] × 100.

Bacteria viability without samples is expressed as follows: 100% in the bacterial viability plot. The mean standard deviation is expressed as relative standard deviation (RSD).

#### Cytotoxicity Test

Mouse embryonic fibroblast NIH-3T3 cells were cultured in Dulbecco’s modified Eagle’s medium (DMEM) supplemented with 10% fetal bovine serum (FBS) (CB_93061524 purchased from Sigma Aldrich), containing penicillin and streptomycin antibiotics (100 μg/ml each) at 37°C in a humidified atmosphere containing 5% CO_2_. The cells were seeded in 6-mm-wide Petri dishes and directly co-treated with 1 mg of the investigated samples. After 24-h exposure time, the medium was removed and replaced with fresh medium containing 0.5 mg/ml thiazolyl blue tetrazolium bromide [also known as MTT or 3-(4,5-dimethyl-2-thiazolyl)-2,5-diphenyl-2H-tetrazolium bromide, purity ≥97.5% (HPLC) and produced by Sigma Aldrich] at 37°C for 4 h. Then, this culture medium was removed, and dimethylsulfoxide (DMSO) was added to 96-well plates. Optical density of the solution was measured at 570 nm with a microplate reader to release the colored product into the solution.

#### Life Cycle Assessment Analysis

LCA is a methodology for assessing environmental impacts associated with the different stages of the life cycle of a commercial product, process, or service (Guinee, 2002). Procedures for conducting LCA are included in the 14,040 series of environmental management standards (ISO 14040, 2006).

The goal of LCA in this study was to evaluate the environmental footprint of AAFs at the product stage, meaning that the “cradle-to-gate” LCA approach was used. This approach takes into account the following: 1) the supply of raw materials, 2) their transport to the product production site, and 3) the manufacturing of the product (European Commission - Joint Research Centre. Institute for Environment and Sustainability, 2010). The functional unit was the production of 1 kg of AAF.

An LCA study involves a thorough inventory of energy and materials that are required across the industry value chain of the product (so-called inventory analysis) and calculates the corresponding emissions to the environment (Horne et al., 2009). LCA, thus, assesses cumulative potential environmental impacts (so-called impact assessment). GaBi 10.0 software (Sphera Solutions GmbH, Berlin, Germany) was used for the performance of the LCA. GaBi uses different databases, and the presented work was done using ecoinvent (version 3.6) database. However, datasets of some chemicals/raw materials are not available in the ecoinvent database. For this reason, data inventory of potassium silicate and metakaolin was taken from literature (see Kvočka et al., 2020).

## Results

### Integrity Test

**FIGURE 1:**
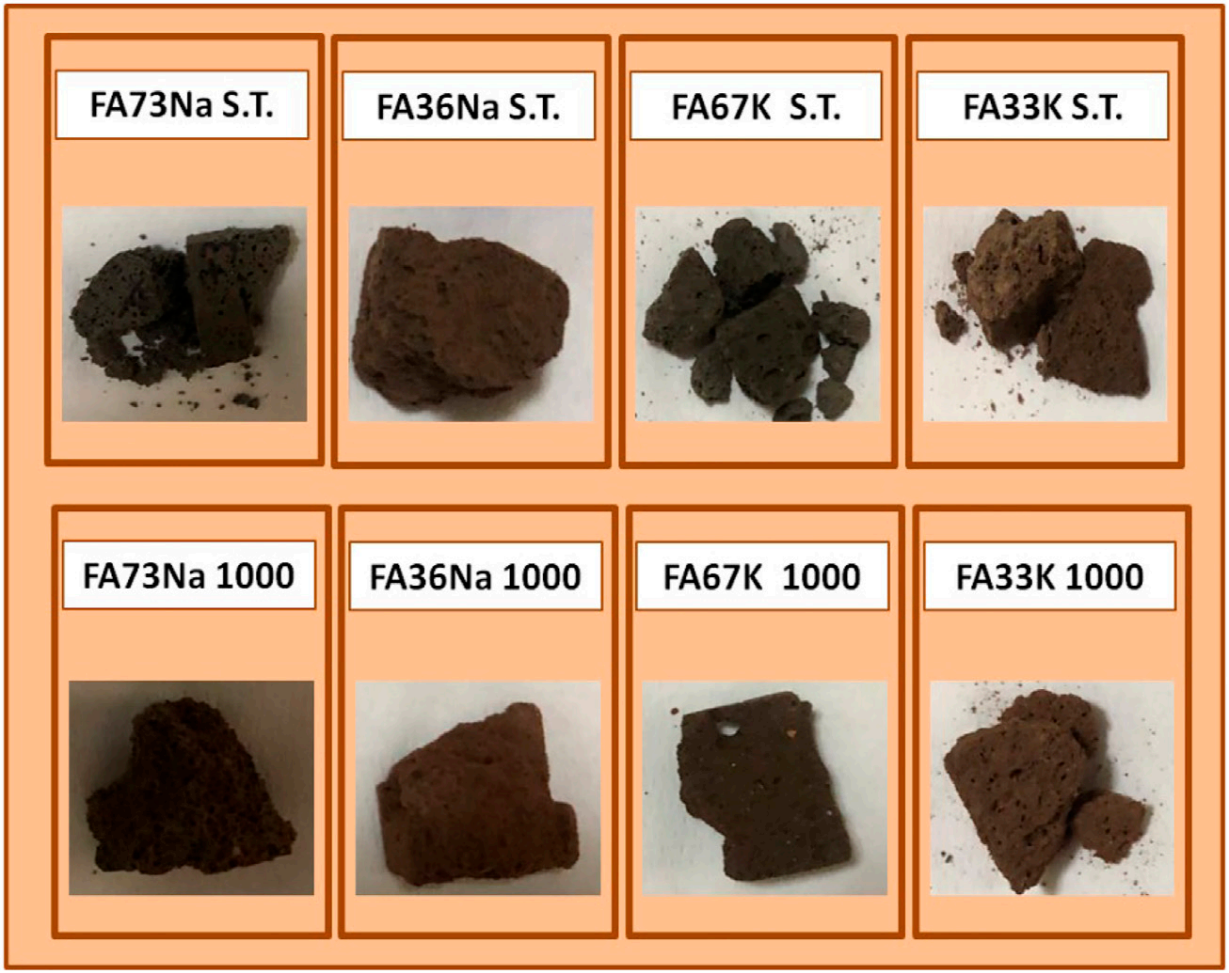
Sample images after the integrity test.

Figure 1 shows the images of the samples after the integrity test. Briefly, FA73Na S.T. was not finger pressure resistant after the test, as it broke down in two parts and lost some pieces. The water of this sample had a pH = 10.94 and was colorless and transparent with some little fragments. On the contrary FA73Na 1,000 was very finger pressure resistant. Water to which sample was exposed was visually very clear with no fragments. Final water pH was 8.7. FA36Na S.T. was also finger pressure resistant. The water of this test was limpid with few microscopic particles and recorded a pH equal to 10.82. Also, FA36Na 1,000 possessed the same feature as FA36Na S.T., but the final pH was 8.72. FA67K S.T. was not finger pressure resistant (it broke in many parts). The water was clear with some fragments. The final pH was 10.86. On the contrary, FA67K 1,000 was very resistant to finger pressure. The water was very clear and had a pH = 8.6. Finally, FA33K S.T. was finger pressure resistant, but it broke in many parts after dropping on the ground. The water was limpid with some fragments. The water pH was 10.73. Also, FA33K 1,000 was finger pressure resistant and broke in three parts after dropping on the ground. The water was clear with no fragments. The pH was 9.31.

### pH and Ionic Conductivity

**FIGURE 2:**
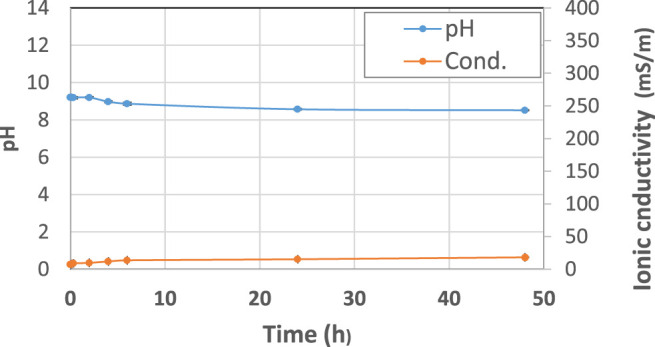
pH and ionic conductivity after immersion in water of metakaolin (MK) precursor from *t*_1_ = 0 h to *t*_9_ = 48 h. A blue line indicates pH and an orange line indicates conductivity changes over time. The error bars are reported as min and max values recorded in three different tests, being approx. 2% on pH and 8% on ionic conductivity.

**FIGURE 3:**
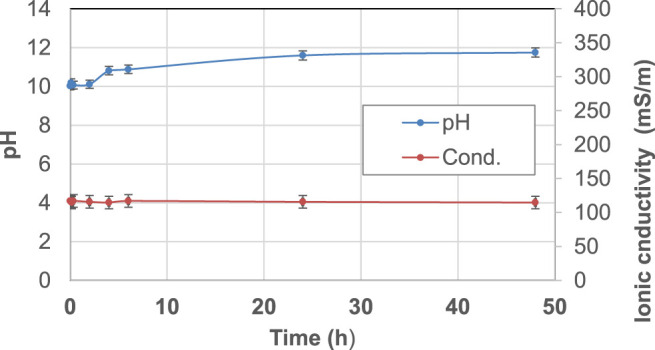
pH and ionic conductivity after immersion in water of FA precursor from *t*_1_ = 0 h to *t*_9_ = 48 h. A blue line indicates pH and an orange line indicates conductivity changes over time.

The starting raw materials present two typical behaviors: metakaolin (Figure 2) is an inert powder with non-soluble salts and has a pH slightly higher than neutral and very low ionic conductivity, as expected. Fly ash (Figure 3) exhibits an alkaline pH and a medium-low ionic conductivity, indicating a very limited presence of soluble salts.

**FIGURE 4:**
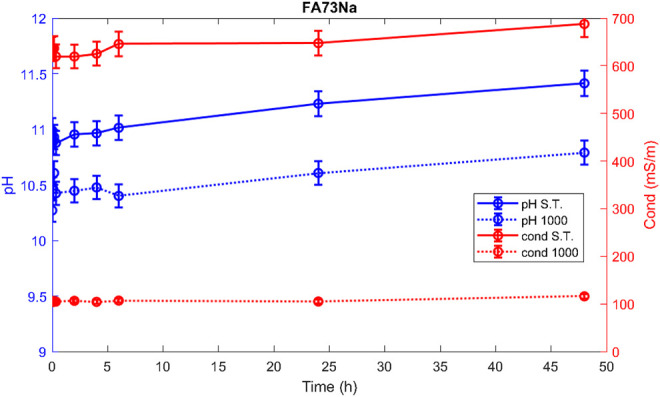
pH and conductivity from *t*_1_ = 0 h to *t*_9_ = 48 h of FA73Na S.T. and FA73Na 1,000.

**FIGURE 5:**
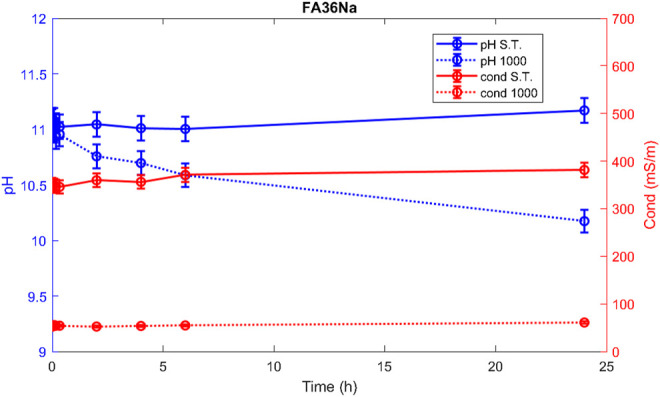
pH and conductivity from *t*_1_ = 0 h to *t*_9_ = 48 h of FA36Na S.T. and FA36Na 1,000.

**FIGURE 6:**
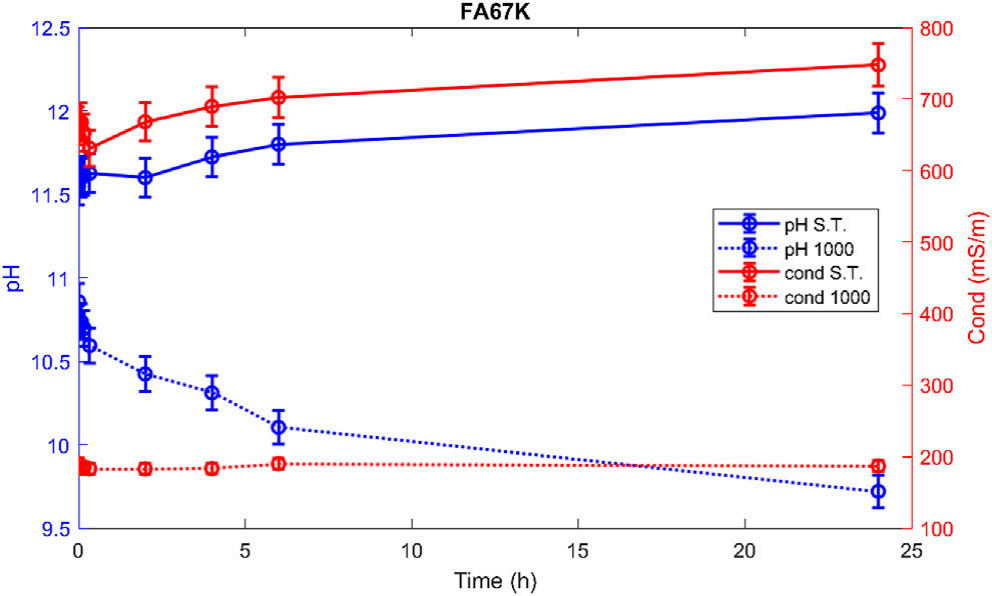
pH and conductivity from *t*_1_ = 0 h to *t*_9_ = 48 h of FA67K S.T. and FA67K 1,000.

**FIGURE 7:**
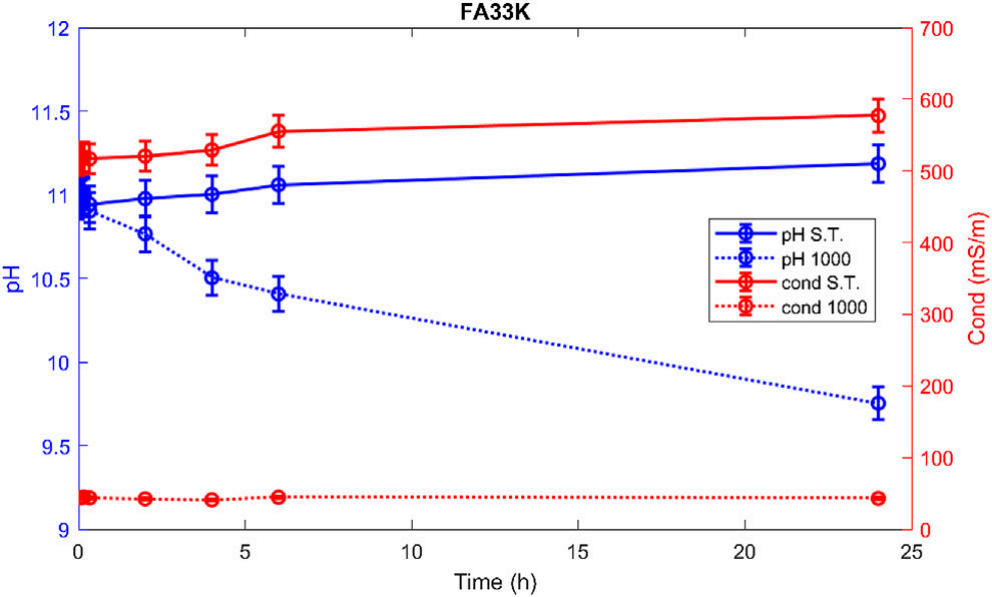
pH and conductivity from *t*_1_ = 0 h to *t*_9_ = 48 h of FA33K S.T. and FA33K 1,000.

Hereafter, the pH and ionic conductivity plots for the AAM and their corresponding fired material are reported (Figures 4–7). The values of the pH decrease after firing, still indicating reactive materials when the pH value is positioned above 8. Ionic conductivity is reduced drastically with firing, still remaining higher than MK, used as a reference for inert material.

The trend towards a continuous dissolution is more evident for the ionic conductivity plots, rather than for the pH. The tendency to increase conductivity with time is more common for the S.T. samples, rather than for the fired ones. The K-activated specimens present a higher tendency to dissolve with time and higher values of ionic conductivity.

### Leaching Test

In order to evaluate the results obtained during the leaching tests, leachate from samples (MK, FA, FA33K S.T., FA36Na S.T., FA67K S.T., and FA67K 1,000) neutralized in concentrated acid were analyzed by ICP-MS for the trace element content. The content in heavy metal is reported in Table 2.

**TABLE 2:** The concentration (mg/l) of the content in heavy metal in the samples.

ppm	Fe	V	Cr	Mn	Zn	Ba
MK	15–64	0.1–0.2	<0.1	0.1–2	0.1–0.2	0.1–0.7
FA	14–87	0.1–0.3	0.1–0.2	0.0–2	0.0–0.4	0.0–0.8
FA33K S.T.	29–35	0.1–0.2	<0.1	0.6–0.7	0.1–0.2	0.1–0.2
FA36Na S.T.	37–41	0.1–0.2	<0.1	0.8–0.9	0.1	0.3
FA67K S.T.	41–49	0.1–0.2	<0.1	1.3–1.4	0.2–0.3	0.3–0.4
FA67K 1,000	46–52	0.1–0.2	<0.1	1.4–1.7	0.2–0.3	0.3

The following elements were found in amounts lower than 0.1 ppm: Co, Ni, Cu, As, Se, Ag, Cd, Sb, Tl, Pb, and Be.

The lower limit of detection is 2 μg/l, while the upper limit of detection is 500 μg/l. When solutions were too concentrated, dilutions to 1:100 or 1: 1,000 were operated. Due to sample homogeneity and preparation procedure, the error affecting these results is around 30%.

For the sake of comparison, the leaching behavior of the two starting materials, MK and FA, was also tested (Table 2). Release of V and Mn was not detected.

The leaching values were compared with EU regulation limits given in the Waste Framework Directive: for inert waste and for non-hazardous waste. Leaching value for Cr in the FA is higher than the law limit for inert waste (indicated in bold in Table 3). For samples FA36Na S.T. and FA67K 1,000, the values of Cr leaching results are higher than the Cr content in the corresponding AAF. Considering an error of 30%, all these values are in the same order of magnitude, indicating that all the Cr content in the AAF was resealed in solution. FA and two of the alkali-activated formulations, samples FA36Na S.T. and FA67K 1,000, are not considered inert waste, due to Cr release in water, while all the other compositions can be considered inert wastes since Zn and Ba release in water are well below law limit. FA and two of the alkali-activated formulations, samples FA36Na S.T. and FA67K 1,000, can be classified as non-hazardous.

**TABLE 3:** The concentration (mg/l) of leached heavy metal ions from the AAF and raw materials.

ppm	Cr	Zn	Ba
FA	0.06	0.00	0.08
MK	0.03	0.00	0.01
FA36Na S.T.	0.16	0.01	0.01
FA36Na 1,000	0.03	0.01	0.02
FA73Na S.T.	0.04	0.01	0.01
FA73Na 1,000	0.07	0.00	0.06
FA67K S.T.	0.1	0.03	0.01
FA67K 1000	0.14	0.01	0.22
FA33K S.T.	0.1	0.03	0.01
FA33K 1,000	0.04	0.01	0.06
Law limit—inert waste^a^	0.05	0.4	2
Law limit—non-hazardous waste^a^	1	5	10

a EU directive—WFD.

The lower limit of detection is 2 μg/l, while the upper limit of detection is 500 μg/l. When solutions were too concentrated, dilutions to 1:100 or 1: 1,000 were operated. Due to sample homogeneity and preparation procedure, the error affecting these results is around 30%.

### Antibacterial Activity

**FIGURE 8:**
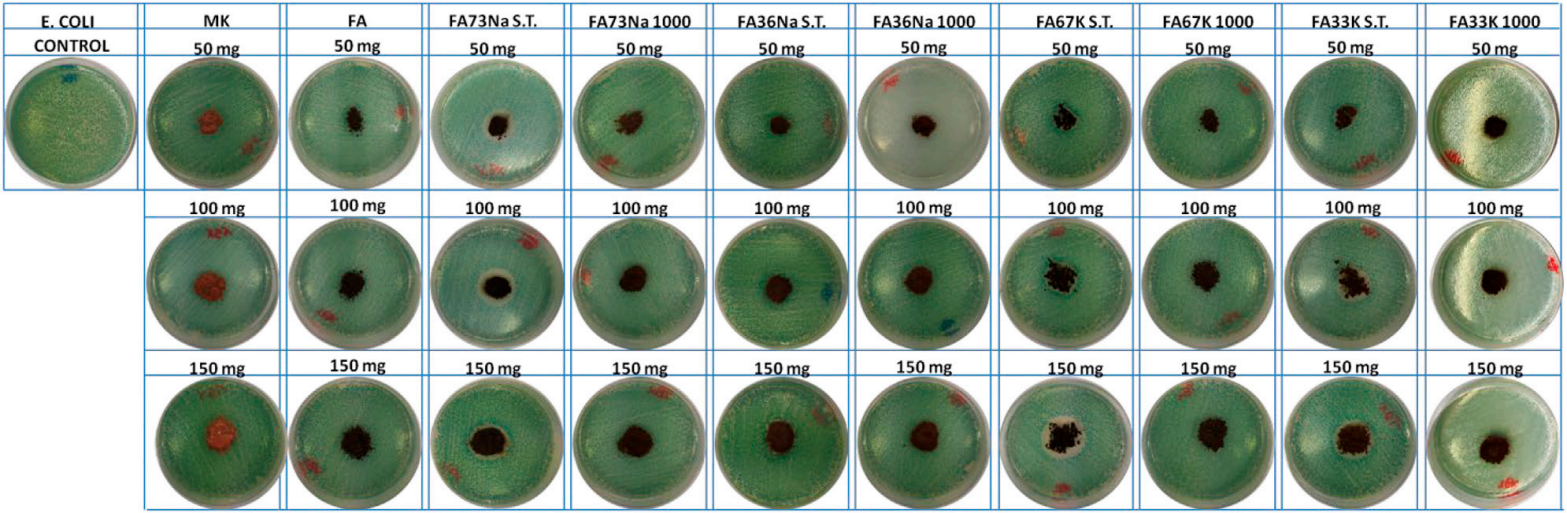
*E. coli* inhibition halo images of the samples.

**FIGURE 9:**
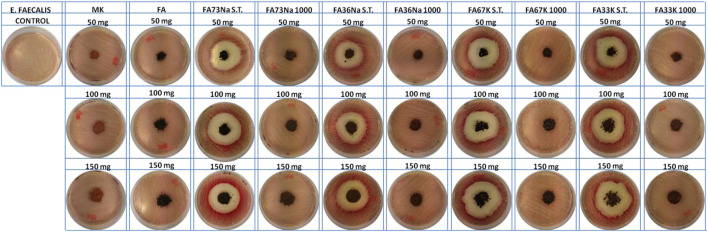
*E. faecalis* inhibition halo images of the samples.

Figures 8 and 9 show the *E. coli* and *E. faecalis* growth in the absence (control sample) and the presence of MK, FA, and the AAM. The bacterial activity has been evaluated measuring the lethal efficacy of the consolidated AAFs in contact with cultural broth of gram-positive and gram-negative families grown in Petri dishes. The chromogenic substrate adopted for the *E. coli* culture has a homogeneous green color in the Petri dish used as reference, while it presents a transparent colorless halo around the powdery specimen, when present (Figure 8). Similar considerations can be done for the chromogenic reddish substrate used for *E. faecalis*, where the colorless area, or inhibition halo, is more evident (Figure 9). As described in the *Antibacterial Activity* section, the diameter of inhibition halos (IDs) in relation to Petri dish diameter was calculated to express the bacterial viability (in percentage). It can be noticed that generally all the samples have no high antibacterial properties on *E. coli* (Figure 8); instead, the S.T. samples have high antibacterial properties on *E. faecalis*. FA73Na S.T., FA67K S.T., and FA33K S.T. (Table 4) have higher reduction of the bacterial *E. coli* viability with respect to the other samples and to the precursors (MK and FA, Table 4). FA73Na S.T., FA36Na S.T., FA67K S.T., and FA33K S.T. possess an increased reduction of bacterial *E. faecalis* viability (Table 4). Generally, it can be noticed that comparing the reduction of bacterial viability, an increase of the sample quantities (50, 100, and 150 mg, respectively) induces a viability reduction (Table 4).

**TABLE 4:** The viability of *E. coli* and *E. faecalis* bacteria in the presence of all the samples tested singularly in different amounts (50, 100, and 150 mg).

Sample	Bacterial viability					
	*E. coli*			*E. faecalis*		
	50 mg	100 mg	150 mg	50 mg	100 mg	150 mg
MK	83% ± 6%	78% ± 6%	76% ± 5%	86% ± 2%	80% ± 6%	77% ± 4%
FA	86% ± 6%	80% ± 6%	78% ± 7%	85 ± 5%	80% ± 6%	78% ± 7%
FA36Na S.T.	85% ± 4%	80% ± 5%	77% ± 5%	54% ± 2%	48% ± 3%	48% ± 3%
Fa36Na 1,000	83% ± 6%	80% ± 5%	79% ± 4%	85% ± 8%	82% ± 4%	79% ± 2%
FA73Na S.T.	74% ± 4%	72% ± 5%	68% ± 3%	51% ± 3%	49% ± 4%	50% ± 2%
FA73Na 1,000	82% ± 5%	81% ± 8%	76% ± 8%	84% ± 7%	81% ± 3%	78% ± 7%
FA67K S.T.	80% ± 5%	74% ± 5%	67% ± 4%	49% ± 4%	45% ± 7%	38% ± 4%
FA67K 1000	86% ± 6%	78% ± 6%	79% ± 7%	84% ± 3%	80% ± 4%	79% ± 6%
FA33K S.T.	80% ± 6%	73% ± 2%	66% ± 5%	47% ± 5%	45% ± 3%	40% ± 4%
FA33K 1000	83% ± 4%	81% ± 5%	79% ± 4%	83% ± 6%	80% ± 6%	78% ± 3%

### Cytotoxicity

**FIGURE 10:**
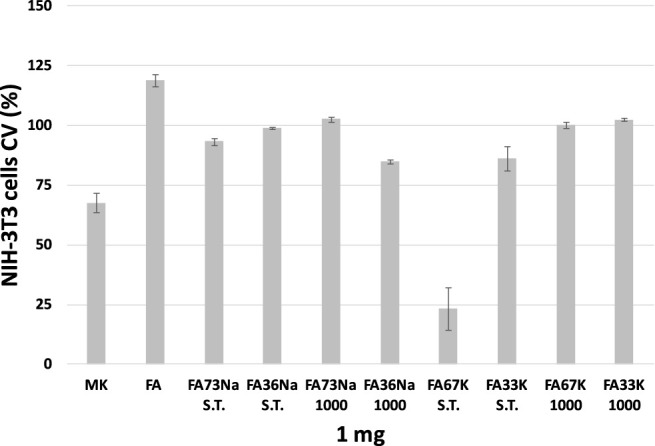
MTT data expressed as cell viability (CV, %) ± SD of three independent measurements.

**FIGURE 11:**
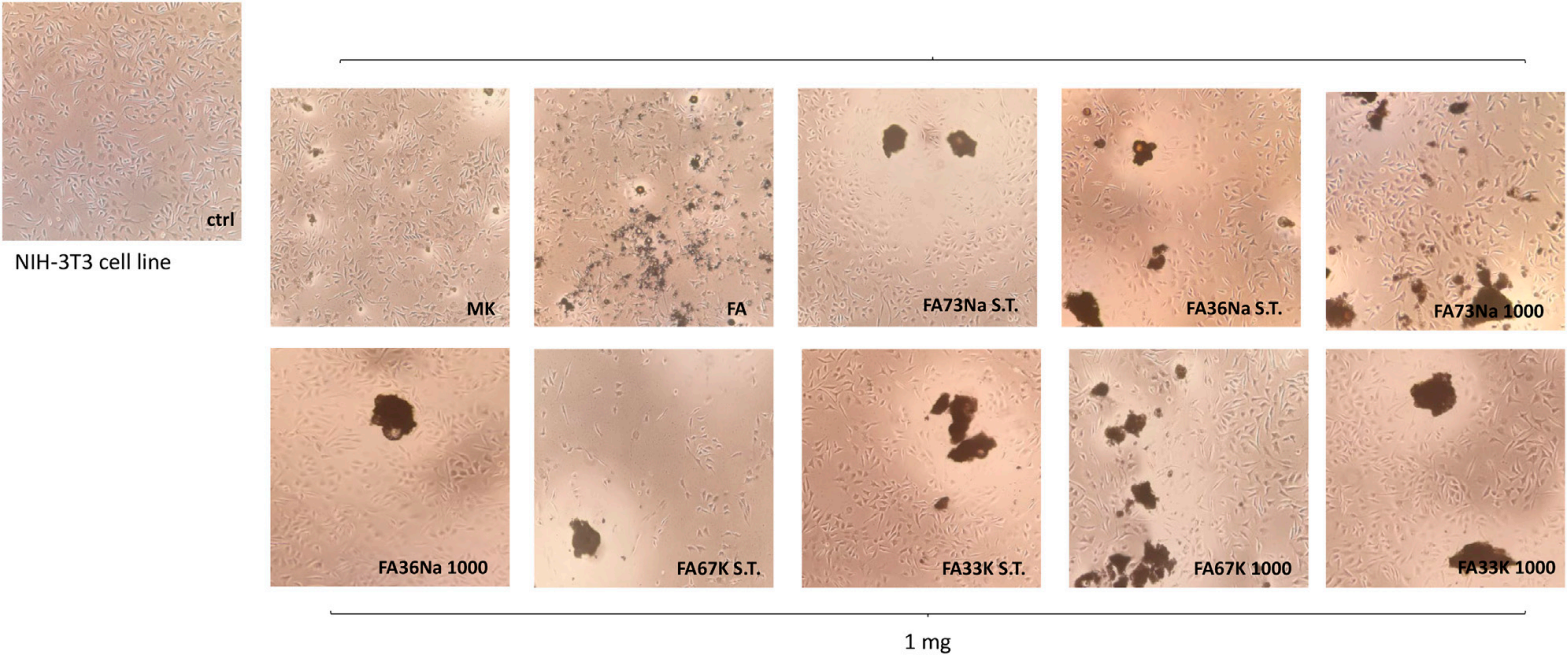
Representative images were acquired by inverted-phase contrast brightfield Zeiss Primovert microscope. Ctrl = untreated cells.

Cytotoxicity was evaluated towards the NIH-3T3 fibroblast cell line, which was placed in direct contact with the samples in the culture medium. Indirect test of the cytotoxicity of AAFs was measured by counting the cell growth (Figure 10) as well as by the observation of the morphological transformations that occurred to the fibroblasts (Figure 11). In the first case, the antiproliferative activity acquired by the MTT test performed after 24-h exposure time is expressed as the number of cells that survived or even proliferated (cellular viability, CV) in the presence of the test material. The lower the value reported in the plot of Figure 10, the stronger and the more acute is the cytotoxic effect manifested by that specific formulation of AAF. The data acquired highlighted that FA67K S.T. exhibits the higher toxicity being able to inhibit NIH 3T3 cell growth by 76.7%, whereas MK sample decreased redox mitochondrial activity by 32.4% (Figure 10). FA36Na 1,000 and Fa33K S.T., as well as FA73Na 1,000, exerted a weak inhibitory activity, in any case less than 25%. All the other samples showed to preserve cell growth, also maintaining NIH 3T3 morphological features (Figure 11). Furthermore, it was observed that the FA sample induced an increase in redox mitochondrial activity of the fibroblast cells equal to 19%.

### Life Cycle Assessment

Environmental performance of AAF based on Na activators and K activators is presented in the form of various environmental and human impact indicators related with manufacturing of the foams (Table 5). As evident from Table 5, manufacturing of AAF based on K activators yields in general lower environmental impacts than manufacturing of the foams based on Na activators. Moreover, replacing a certain amount of FA (FA73Na S.T.) with metakaolin (FA36Na S.T.) results in increase of the impacts.

**TABLE 5:** The environmental performance of AAF taking into account the CML 2001 impact assessment method.

Impact category	Unit	FA33K S.T.	F67K S.T.	FA36Na S.T.	FA73Na S.T.	Additional firing at 1,000°C
Abiotic depletion of elements	kg Sb eq	6.63E−05	5.72E−05	1.11E−04	7.39E−05	6.47E−05
Abiotic depletion of fossil	MJ	116.3	113.7	119.3	119.7	117.0
Acidification potential	kg SO_2_ eq	0.151	0.146	0.153	0.153	0.148
Eutrophication potential	kg PO_4_ eq	5.42E−02	5.30E−02	5.46E−02	5.47E−02	5.36E−02
Freshwater aquatic ecotoxicity pot	kg DCB eq	14.0	13.9	14.5	14.2	14.0
Global warming potential	kg CO_2_ eq	9.8	9.5	10.1	10.2	9.9
Global warming potential, excluding biogen C	kg CO_2_ eq	9.7	9.4	10.0	10.0	9.8
Human toxicity potential	kg DCB eq	10.7	10.5	11.7	11.1	11.0
Marine aquatic ecotoxicity pot	kg DCB eq	27,659.3	27,324.9	28,395.8	28,130.77	27,796.38
Ozone layer depletion potential	kg R11 eq	7.78E−07	7.53E−07	9.14E−07	9.16E−07	8.91E−07
Photochem. ozone creation potential	kg Ethene eq	6.61E−03	6.38E−03	6.71E−03	0.00671	0.006483
Terrestrial ecotoxicity potential	kg DCB eq	0.193	0.191	0.199	0.198353	0.19648

## Discussion

**FIGURE 12:**
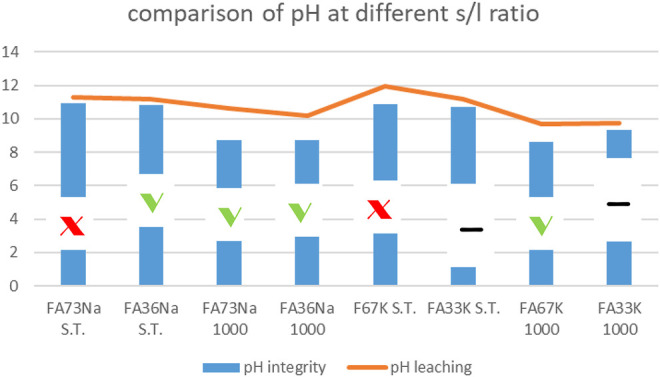
pH values versus integrity test response of the AAFs: specimens that passed the test are indicated with green thick. Samples that did not pass are indicated with red cross. The other two samples had an intermediate, yet unacceptable behavior. For error bars, see Figures 2–7 or Figure 13.

Given that fly ash is a residual material, an environmental study has been carried out to characterize the product more completely in order to make a better evaluation about its possible uses as construction materials. In particular, the influence of low-calcium fly ash products on life activities of flora and fauna has already been investigated (Kozhukhova et al., 2019), observing a negative effect onto the ecosystem due to release of the ions such as Na^+^, Cl^–^, and SO_3_^2–^ as well as CaO. Nevertheless, the use of fly ash as a major component in geopolymer binders assumes the physical–chemical encapsulation with consequent prevention of the release of toxic cations and ions into the environment (Kozhukhova et al., 2015). With this objective in mind, we started to evaluate the consolidation step for the alkali activation process by investigating the integrity in water of the hardened AAFs. In more details, the integrity test indicates that the investigated AAFs have different resistance toward immersion in water for 24 h. The plot reported on Figure 12 compares the pH values recorded during the following:

**FIGURE 13:**
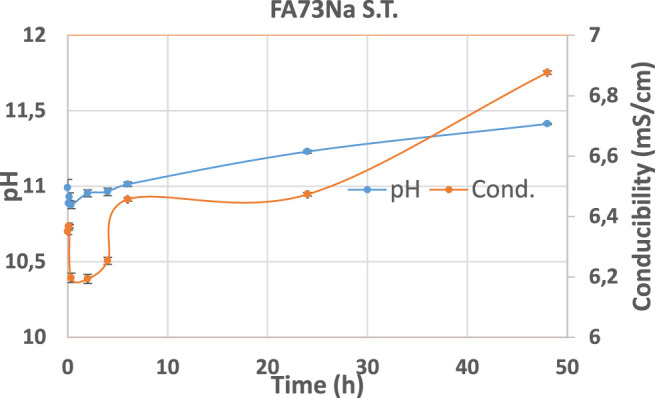
pH and ionic conductivity of FA73Na S. T. in an enlarged view.

i) Integrity tests, with solid-to-water ratio = 1:100

ii) Leaching tests, with solid-to-water ratio = 1:10.

During leaching, the pH values are higher; then, during the integrity tests, due to higher amount of solid in the same amount of water, such very small difference between the two tests was expected. In Figure 12, the –OH groups have left the material at 1,000°C due to the dehydroxylation process, thus lowering the pH for those compositions receiving the thermal stabilization treatment (FA73Na 1,000, FA36Na 1,000, and FA67K 1,000) (Kozhukhova et al., 2020). The integrity tests indicated the formulation that passed the water immersion: four samples mostly activated with NaOH. It is unexpected that thermal treatment at 1,000°C does not always produce better samples, see the FA33K 1,000 case, but the thermal instability of the CASH phase as a consequence of the CaO presence (12.38 wt%) in the AAFs could be responsible.

For some compositions, the instability in the pH and ionic conductivity behaviors of the first period of test is more evident in the enlarged plot of Figure 12, where the error bars also become visible. After 24 h of testing, the trends become more linear.

**FIGURE 14:**
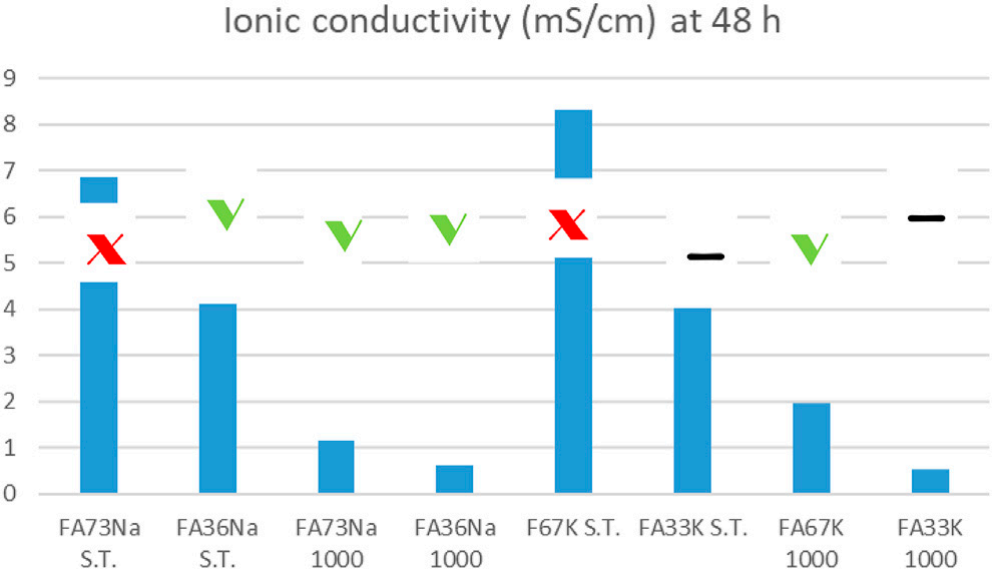
Ionic conductivity values versus integrity test response of the AAM: specimens that passed the test are indicated with green thick. Samples that failed are indicated with red cross. The other two samples had intermediate, yet unacceptable behavior. For error bars, see Figures 2–7 or Figure 13.

Comparing the results of the integrity test with the values of the ionic conductivity (recorded at 48 h), the chemical instability of the K-activated materials becomes more visible with respect to the Na-activated one (Figure 14).

Low ionic conductivity (sample FA33K 1,000) and satisfactory integrity test are indications of a good chemical stability.

The leaching study involved subjecting the material to one of the most commonly used tests for monolith samples in the waste management field in Europe (EN 12457 European standard “Characterization of waste–Leaching–Compliance test for leaching of granular waste materials and sludge”). The leachates were measured using ICP-MS equipment (Tables 2 and 3), and results indicated that neither of the hardened AAM nor precursor can be classified as hazardous according to WFD criteria for hazardous waste. All of the AAFs can be classified as inert, with the exception for FA36Na S.T. and FA67K 1,000. Differently from published results on FA-based AAMs, focused on fungicidal properties of some FA products (Vasilenko et al., 2020), we investigated the effect on bacteria, either gram-positive (*E. faecalis*) or gram-negative (*E. coli*). Experimentally, we demonstrated that there is no obvious negative effect on bacterial growth, thus declaring a safe coexistence of the developed geopolymers with the ecosystem. Specifically, the highest impact on bacterial colonies was found for the following: FA73Na S.T., FA36Na S.T., FA67K S.T., and FA33K S.T., which induced the reduction of bacterial *E. faecalis* viability. The higher toxicity was exhibited by FA67K S.T.; this specimen is the AAF with the higher pH and the higher degree of dissolution in water, also being able to inhibit NIH 3T3 cell growth at the highest percentages. The toxicity of the other AAFs cannot be correlated with Cr release (higher Cr leaching values were recorded for FA36Na S.T. and FA67K 1,000). An effect of FA was recorded as an increase in redox mitochondrial activity of the fibroblast cells, but this phenomenon was not recorded for the AAMs.

**FIGURE 15:**
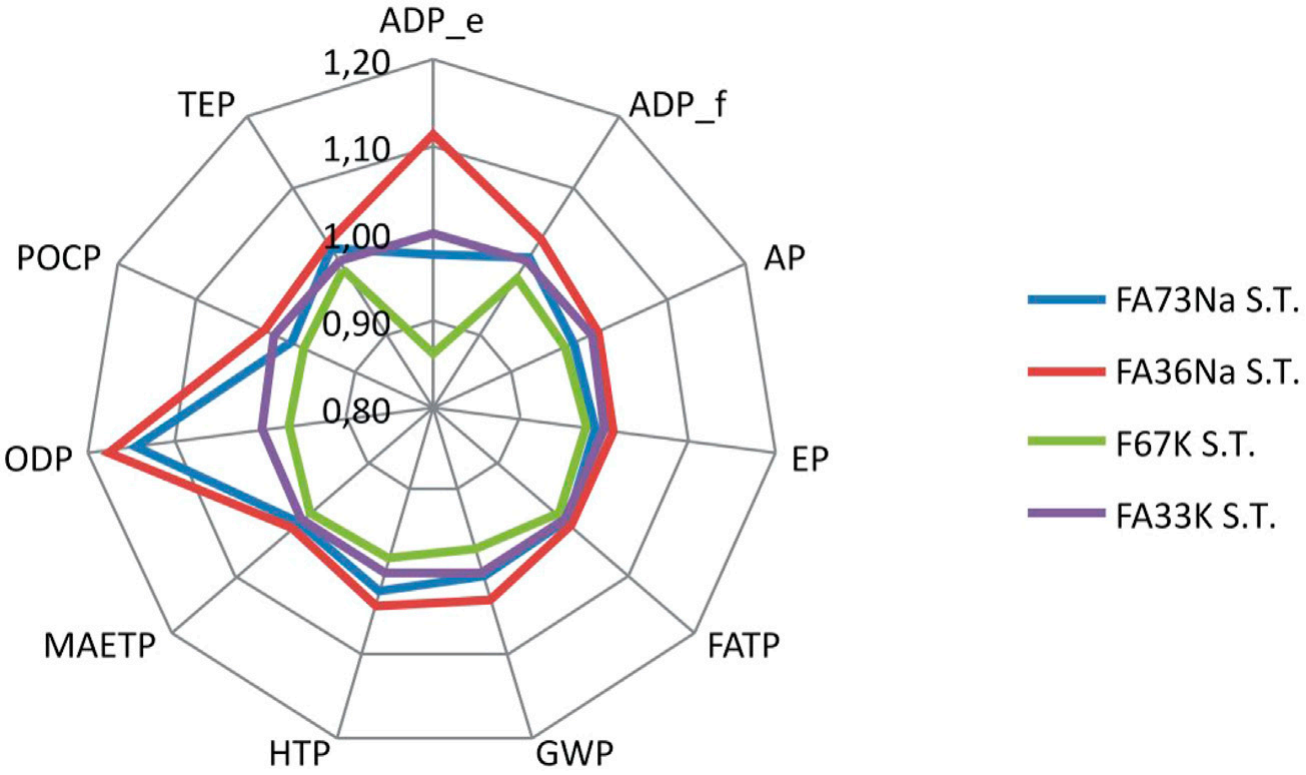
Relative comparison of the environmental footprints of the AAFs manufactured *via* different recipes. AAFs with Na-based activator were set as a reference.

**FIGURE 16:**
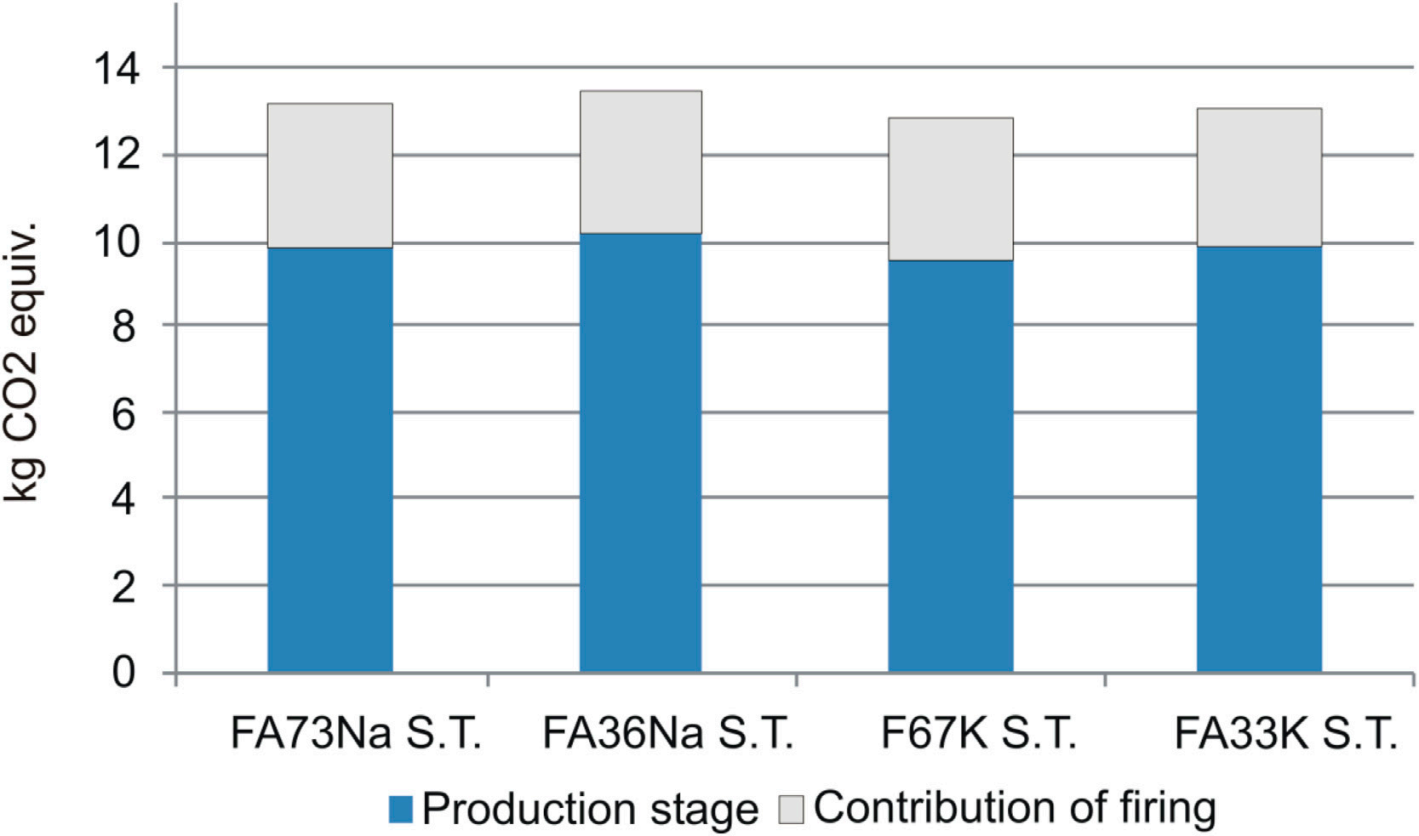
Comparison of global warming potentials of four AAFs.

LCA results have shown that manufacturing of alkali-activated foams based on K activators and full content of fly ash (without metakaolin as a partial substitute material for fly ash) (e.g., recipe F67K S.T.) generate the lowest environmental impacts, compared to other recipes (Figure 15). Taking into account the potential impact on global warming potential (related with greenhouse gas emissions, which are converted to kilogram CO_2_ equivalents), AAF based on Na activators yield around 0.3 kg of CO_2_ equiv. emissions more than AAF based on K activators (Figure 16).

As evident from Figure 16, firing AAF at 1,000°C significantly contributes to the global warming potential of AAF. In such a case, the environmental footprint of the foams increases for around one third (additional 3.3 kg CO_2_ equiv. emissions), compared to the footprint of AAF produced at 70°C (Figure 16).

Replacing some parts of FA (FA73Na S.T. and F67K S.T) with metakaolin (FA36Na S.T. and FA33K S.T.) results in an increase of global warming potential for around 3%, while changing K-based activators (F67K S.T and FA33K S.T.) to Na-based activators (FA73Na S.T. and FA36Na S.T.) increases global warming potential for 4% (taking into account the production stage) (Figure 15).

The main environmental disadvantage associated with partially replacing of FA with metakaolin is related to the potential abiotic depletion of elements, which is increased by 14% (Figure 15). The contribution of metakaolin to the potential abiotic depletion of elements is around 12%–14% (depending on the recipe), while the contribution of fly ash (considered as an industrial by-product) is negligible (less than 0.01%), taking into account the production stage of the alkali-activated foams.

Changing K-based activators to Na-based activators results in a significant increase of impact on abiotic depletion of elements (for 11%) and even more significant increase of impact on ozone layer depletion (for 18%) (Figure 15). Sodium hydroxide yields five times higher impact on ozone layer depletion than potassium hydroxide, comparing the synthesis of the same amount of these two chemicals (ecoinvent v3. 2019). A significant contribution of sodium hydroxide to the impact of ozone layer depletion in case of geopolymer production was also revealed by Passuello et al. (2017). The raw material, which is responsible for increase of the impact on abiotic depletion of elements, is sodium silicate, which is used for production of Na-based alkali-activated foams. Production of sodium silicate is supposed to yield relatively high impact on abiotic depletion of elements, according to ecoinvent information (ecoinvent v3.6, 2019). However, some precaution should be taken into account, as different sodium silicate production routes exist (Fawer et al., 1999).

**FIGURE 17:**
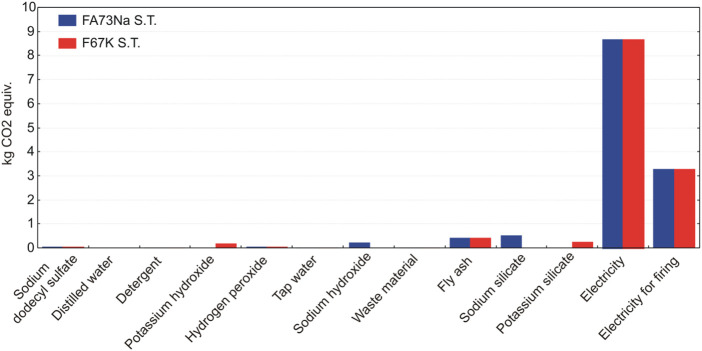
The relative contributions of raw materials, ancillary materials, energy requirements, and waste flows to the AAF with fly ash, taking into account the production process stage.

**FIGURE 18:**
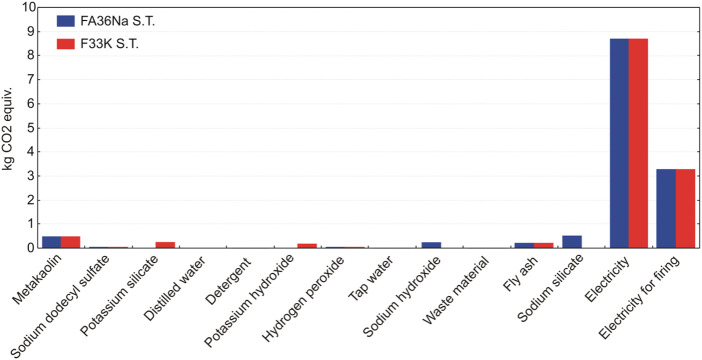
The relative contributions of raw materials, ancillary materials, energy requirements, and waste flows to the AAF with partial replacement of fly ash with metakaolin, taking into account the production process stage.

A contributor analysis also shows that electricity requirements for the manufacturing of the AAF represent the main environmental hot spot, contributing most of the impacts (Figures 17 and 18). In case of firing AAF at 1,000°C, the environmental impacts related with electricity requirement increase by around one third, as evident also from Figure 15.

LCA analysis has identified the major environmental impacts of AAF life cycle production trough different indicators. The main contribution to global warming potential of all other considered impact categories arises from electricity needed for the production (Figures 17 and 18). Moreover, the environmental footprint of the foams increases significantly if they are subjected to the firing up to 1,000°C, due to additional electricity requirements. The environmental benefits and drawbacks will be more clearly seen by 1) expanding the system boundary into the mode, which includes end-of-life options, and 2) comparing AAF to some commercial-based solutions, and both will be the subject of future work.

## Conclusion

AAFs were produced from two precursors, fly ash and metakaolin, by two Na-based and K-based activators. These different foams were not only cured at room temperature but also treated at 1,000°C in order to make them more stable. Previous research has confirmed that their performance would be suitable for application in building and industrial sectors in terms of technical parameters. Given that fly ash is a residual material, in the present study, these foams were also evaluated from an environmental point of view.

Tests and evaluations have shown that both precursors themselves do not exceed limit values for non-hazardous waste, but fly ash slightly exceed the value for Cr and Mo when referred to the limit values for inert waste. After alkali activation in some cases, even in cases of high-temperature-treated samples, Cr and Mo are much above the values for inert and for non-hazardous waste. No specific trend can be observed, and contrary to the expectations, the sample fired at 1,000°C was also not stabilized enough. Alkali activation can thus degrade environmental parameters, as precursors are exposed to an alkaline medium that leaches out a higher value of certain heavy metals, in our case Mo and also Cr to a certain extent, which is especially evident in the case of FA67K, fired at 1,000°C. This does not correspond, contrary to the expectation, to the pH values of leachate, and it might be a result of the non-optimal design of mixture in terms of precursor-to-activator ratio. Regarding antibacterial properties, it can be noticed that in general, all the samples have no antibacterial properties on *E. coli*, while samples prepared at room temperature have higher antibacterial properties on *E. faecalis* in comparison with samples fired at 1,000°C. Similarly, in cytotoxicity test, these samples, especially the one with only fly ash as activator (FA67K S.T.), inhibit NIH 3T3 cell growth most evidently, which corresponds to the higher pH and higher dissolution of these samples in water. For precursor fly ash itself, an increase in redox mitochondrial activity of the fibroblast cells was noticed, but this phenomenon was not recorded for the AAF samples, confirming the encapsulation of toxic ions in the fly ash matrix. However, bacterial growth can cause biocorrosion and durability issues of the developed AAFs; thus a long-term study is here suggested.

Based on the results obtained in the present paper it is of extreme importance to follow environmental parameters, at least in terms of leaching analysis, since alkali activation can significantly change the properties of precursors in this sense. This especially could be an issue when mixtures are not optimally designed, which can easily happen when working with waste materials, not being constantly monitored.

In the LCA analysis, two significant conclusions can be drawn; the first one is that the use of Na activator increases environmental performance of the alkali-activated foams compared to the K-based activators, and as expected, high-temperature treatment increased the environmental footprint by about one third. Comparison to the products from virgin resources also shows significant benefits in decreased abiotic depletion potential when fly ash is used instead of metakaolin.

The products developed in the present work guarantee a low toxicity level assessed through leaching, antibacterial, and cellular viability studies, and they also show environmental benefits in terms of LCA indicators.

## Data Availability

The original contributions presented in the study are included in the article/Supplementary Material; further inquiries can be directed to the corresponding author.
